# The efficacy of EndoActivator, passive ultrasonic irrigation, and Ultra X in removing calcium hydroxide from root canals: an in-vitro study

**DOI:** 10.1186/s12903-022-02626-z

**Published:** 2022-12-03

**Authors:** Alireza Adl, Alireza Razavian, Fateme Eskandari

**Affiliations:** 1 Department of Endodontics, Oral and Dental Disease Research Center, School of Dentistry, Shiraz University of Medical Sciences, Shiraz, Iran; 2 Department of Endodontics, School of Dentistry, Shiraz University of Medical Sciences, Shiraz, Iran; 3 School of Dentistry, Shiraz University of Medical Sciences, Ghasrdasht Street, Shiraz, 71956-15878 Iran

**Keywords:** Calcium hydroxide, EndoActivator, Passive ultrasonic irrigation, Ultra X

## Abstract

**Background:**

This study aimed to compare the efficacy of EndoActivator, passive ultrasonic irrigation, and Ultra X in removing calcium hydroxide from the artificial grooves in root canal walls.

**Methods:**

The root canals of 50 extracted human maxillary incisors were instrumented by using the ProTaper rotary system up to #F4 (size 40/0.06 ProTaper) and the teeth were split longitudinally. Lateral grooves were created in the apical and coronal parts of one half and the middle part of the other half. Calcium hydroxide paste was applied to the grooves and the root halves were reassembled. After seven days, the calcium hydroxide was removed from the canal by using one of the EndoActivator, passive ultrasonic irrigation, and Ultra X devices; one group went without irrigation (control group). The CH remnants in the grooves were scored at 20× magnification. The data were analyzed by using the Kruskal–Wallis, Dunn’s post hoc, and Friedman tests. *P* < 0.05 was considered to be statistically significant.

**Results:**

No statistically significant difference existed among the experimental groups at the coronal and middle grooves (*P* > 0.05). However, Ultra X was significantly more effective than passive ultrasonic irrigation at the apical grooves (*P* = 0.023).

**Conclusion:**

Within the limitations of this study, Ultra X can be reported to remove the calcium hydroxide from the apical third more efficiently than passive ultrasonic irrigation.

## Background

Eradicating or minimizing bacteria and their by-products from root canals and preventing reinfection plays a key role in root canal treatments [1–3]. Optimum disinfection of the root canals is accomplished by mechanical debridement supplemented with root canal irrigants and interappointment medicaments [4, 5]. Calcium hydroxide (CH) stands among the most routinely applied intracanal dressings because of its well-documented advantages including antibacterial activities and various favorable biological properties like biocompatibility, tissue-dissolving ability, and induction of mineralized tissue [6–8]. In addition to dressing of the canals between appointments, CH is also used for a number of other procedures, such as apexification, treatment of root resorption, iatrogenic root perforations, and replanted teeth [9].

The complexity of root canal anatomy makes the complete removal of intracanal medicaments very challenging [10]. The CH residuals have been found to jeopardize the adaption of endodontic sealers to the root canal walls [11] and their penetration into dentinal tubules [12], and consequently compromise the sealing quality of the root filling [13, 14]. Moreover, the CH residuals can chemically react with endodontic sealers and decrease their working time and flow [15]. Therefore, CH should be completely eliminated before obturating the root canal system [16].

Various irrigation solutions and techniques have been investigated for better CH elimination from dentinal walls. Manual instrumentation with a master apical file and copious irrigation seems inadequate for complete dressing removal [17]. To overcome these shortcomings, mechanical agitation of the irrigants has been proposed as a novel technique using sonic and ultrasonic units [10, 18]. One of the most common sonic agitation devices is the EndoActivator system which is comprised of a portable handpiece and three noncutting flexible polymer tips in different sizes. Its design allows safe activation and the production of vigorous intracanal fluid agitation [19, 20]. On the other hand, most ultrasonic devices which are used for passive ultrasonic irrigation (PUI) operate at 25–30 kHz [21]. As the gold standard of irrigant activation [22], PUI transmits the acoustic energy from an oscillating file or smooth wire to an irrigation solution in the root canals [23]. Compared with the traditional methods of root canal irrigation, the classic PUI devices have effectively improved root canal disinfection through the generation of cavitation and acoustic transmission [24–26].

Recently, the Ultra X ultrasonic handpiece has been marketed with a working frequency of 45 kHz. Although the agitation of irrigants with this headpiece can be regarded as a kind of PUI, its higher working frequency than other ultrasonic units may improve its efficacy for cleaning root canals. To the best of the authors’ knowledge, only one study [27] has ever evaluated the effectiveness of Ultra X in eliminating CH from root canals. Therefore, the present study was designed to evaluate the efficacy of Ultra X, the classic PUI system, and EndoActivator in eliminating CH from artificial standardized grooves in the root canal. The null hypothesis was that these devices would not be significantly different.

## Methods

### Sample size calculation

In accordance with previous research [28], a power calculation was conducted by using the chi-square test family and variance statistical test (G*Power 3.1 software; Heinrich Hein University, Dusseldorf, Germany) with α = 0.05 and ß = 0.95, and the sample size was determined to be a minimum of 11 per group.

### Preparation of tooth samples

The study design was approved by the Ethics Committee of Shiraz University of Medical Sciences, Shiraz, Iran (IR.SUMS.DENTAL.REC.1400.038). It was performed in full accordance with ethical principles, including the World Medical Association Declaration of Helsinki (version 2008).

Fifty human maxillary incisors with a minimum length of 18 mm, intact apices, and straight roots were selected from a collection of recently-extracted teeth. The samples were disinfected in 0.5% chloramine-T solution (Merck; Darmstadt, Germany) for 48 h and then stored in distilled water till used. The root canal anatomy was checked on mesiodistal and buccolingual radiographs. Teeth with previous canal treatment, caries, restoration, fractures, cracks, internal/external resorption, and calcification were excluded.

The selected teeth were shortened to achieve a standardized length of 17 mm with a working length of 16 mm. After access cavity preparation, the root canals were instrumented with ProTaper rotary system (Dentsply Tulsa; Switzerland) up to #F4 (size 40/0.06). Between each file, the root canals were irrigated with 2 ml of 2.5% sodium hypochlorite (Chloraxid, Cerkamed, Poland) by using a plastic syringe with a 30-gauge needle (Cerkamed, Poland). Finally, each root canal was rinsed with 5 ml of 17% EDTA for one minute, followed by 5 ml of saline solution.

The samples were fixed in plastic tubes containing silicon impression material (Coltene/Whaledent; Langenau, Germany). Upon removal from the molds, two longitudinal grooves were made at the buccal and palatal surfaces of each tooth by using a diamond disc (Microdont; LDA, Brazil) under water coolant. The roots were then split into halves by using a chisel.

**Fig. 1:**
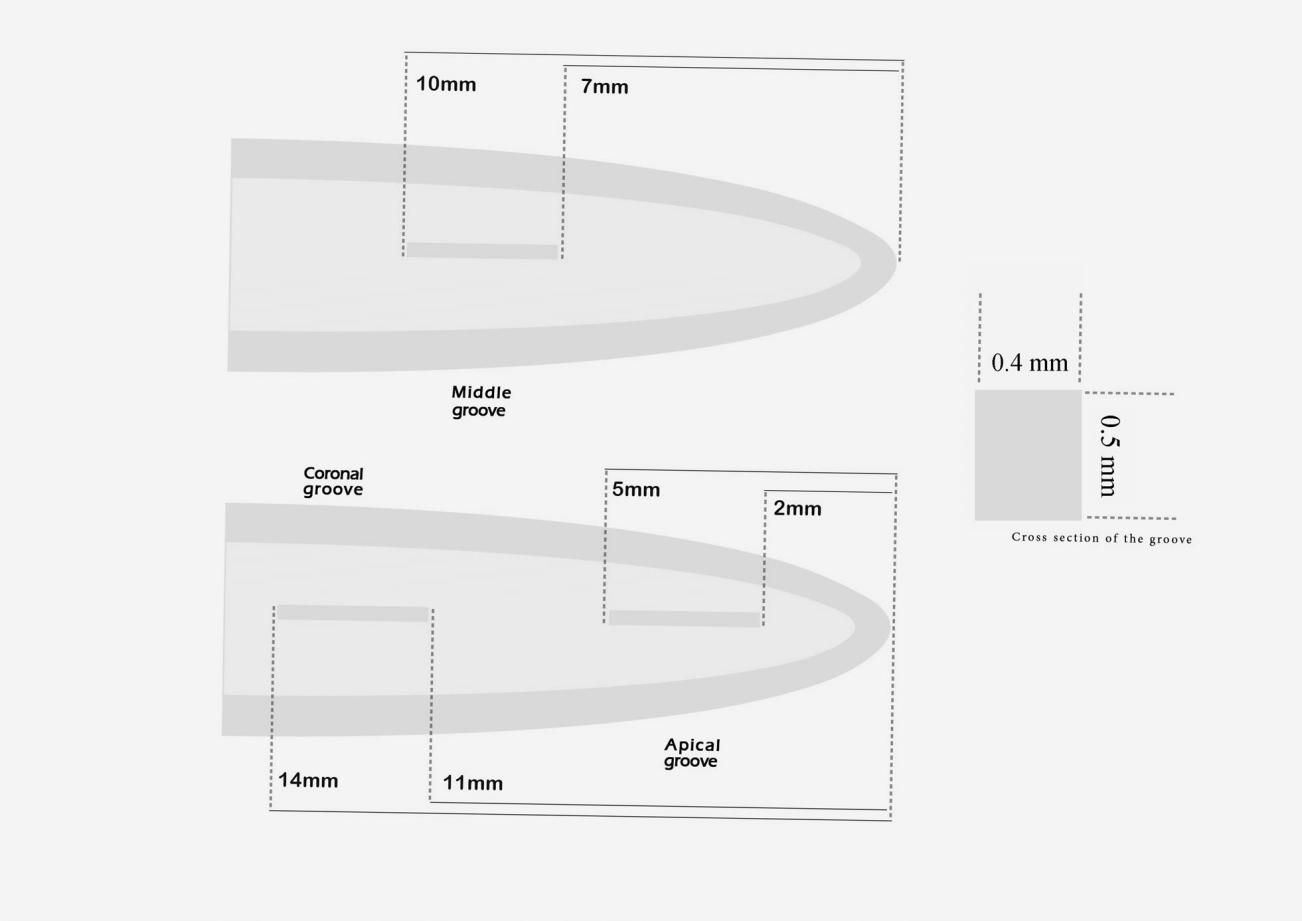
Schematic illustration of size and locations of grooves

The ultrasonic tip was used to create lateral grooves (3 mm long, 0.2 mm wide, and 0.4 mm deep) in the canal side of the halves to simulate unreachable canal recess in the root canal. Then, one-half of each specimen was used to create two grooves at apical (2–5 mm from the apex) and coronal (11–14 mm from the apex) thirds. On the other half, the groove was made at the middle third of the root canal (7–10 mm from the apex) (Fig. 1). Debris was removed from the grooves and root halves by using a toothbrush.

CH powder (Merck; Darmstadt, Germany) was mixed with sterile saline (1:1 ratio) and the grooves were filled by using paper points. The root halves were reassembled with wax. To simulate a closed irrigation system, the apices were also covered with wax and the roots were returned to the molds. The access cavities were sealed with a cotton pellet and Cavizol (Arya Dent; Iran). The samples were incubated at 37 °C in 100% humidity for 7 days. Then, based on the CH removal technique, the teeth were randomly allocated into three experimental groups (n = 15 per group) and a control group (n = 5) where CH was not eliminated.

### Irrigation agitation methods

All root canals were rinsed with 5 mL of 2.5% sodium hypochlorite by a syringe and a 30G needle. Then, the irrigant was activated for 60 s with one of the following three devices:

- A size 25 K ultrasonic file mounted on a piezoelectric handpiece (NSK Various 2; Nakanishi, Tochigi-ken, Japan) with the power setting of 6 (PUI group)
- A tip #25, 0.04 taper EndoActivator system (Dentsply Sirona, New York, USA) set at 10,000 cycles per minute
- An Ultra X (Eighteeth, Changzhou Sifary Medical Technology Co., Ltd, Changzhou City, China) with a flexible X Silver tip (#25, 0.02) according to the manufacturer’s guidelines

All in a length of 1 mm shorter than the working length. Irrigation and activation were repeated twice, resulting in a total of 10 mL of sodium hypochlorite and 2 min of activation. The root canals were ultimately rinsed with 5 mL of distilled water to flush out the remaining sodium hypochlorite. In the control group, CH was not removed from the root canal system.

### CH scores

**Fig. 2:**
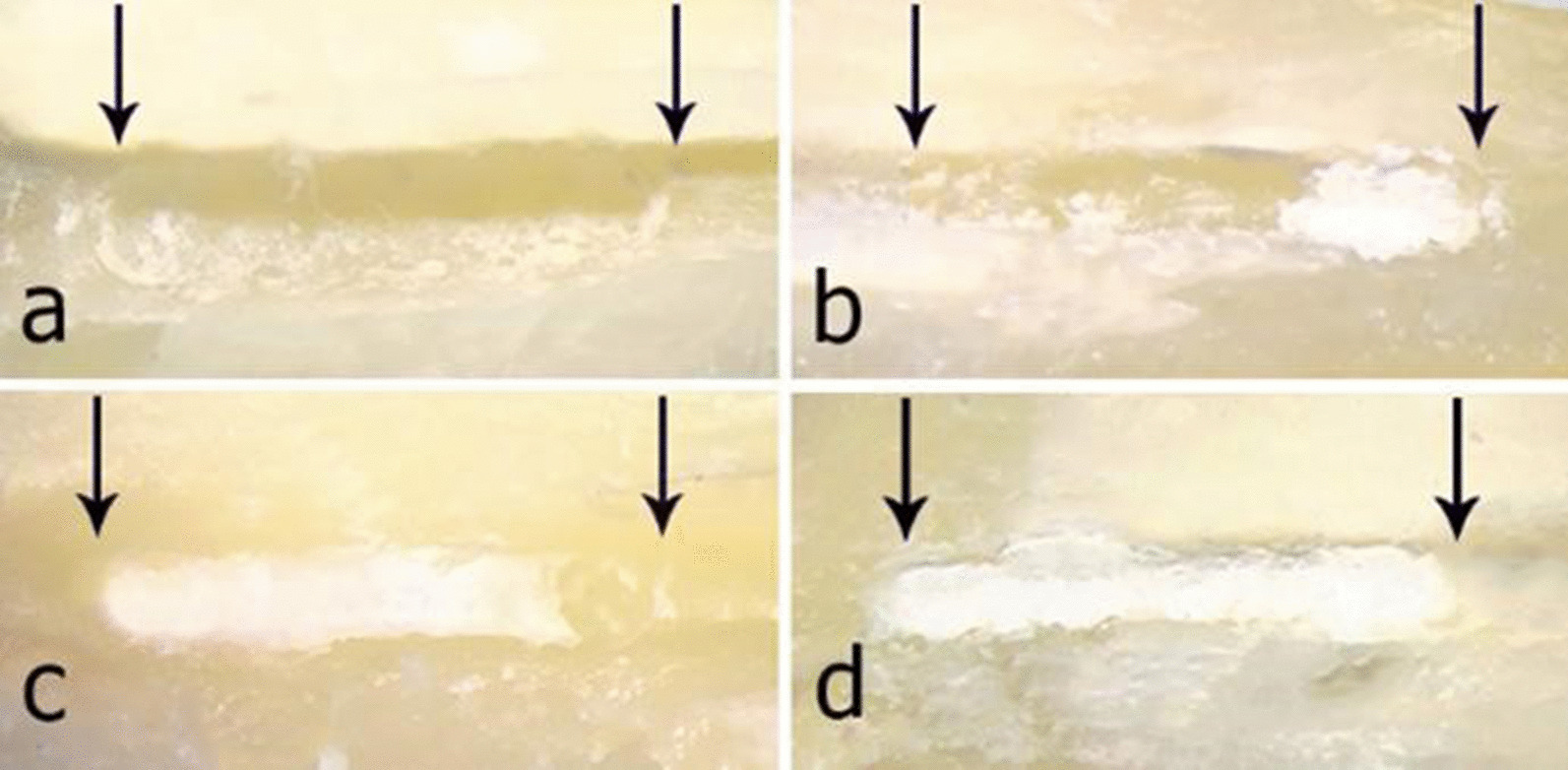
Scoring system for evaluation of CH removal from the artificial grooves. The black arrows indicate the coronal and apical ends of the grooves. **a** Score 0: The groove is empty. **b** Score 1: Less than 50% of the groove is filled with CH. **c** Score 2: More than 50% of the grooves is filled with CH. **d** Score 3: The groove is completely filled with CH

The root canals in all groups were dried by using a paper point (Dentsply Maillefer; Ballaigues, Switzerland) and the root halves were recleaved and inspected by two blinded and calibrated endodontists under a stereo zoom microscope (Best Scope-3060c; China) at 20× magnification. On a 4-grade scoring system (0–3) [29], the CH remnants in the artificial grooves were scored as 0 (empty groove), 1 (< 50% of the groove filled with CH), 2 (> 50% of the groove filled with CH), and 3 (the groove completely filled with CH) (Fig. 2).

### Statistical analysis

Statistical analyses were done by using SPSS software (version 22, SPSS INC., Chicago, IL, USA). The experimental groups were compared regarding the CH scores through Kruskal–Wallis and Dunn’s post hoc tests. The Friedman test was used to compare the CH remnant among the root canal thirds. The level of statistical significance was set at *P* < 0.05 in all tests.

## Results

All the grooves in the control teeth were completely filled with CH (score 3). Table 1 presents the scores of the coronal, middle, and apical grooves in the experimental groups. No statistically significant difference was detected among the experimental groups at the coronal and middle grooves (*P* > 0.05). However, they were significantly different regarding the CH remnants in the apical grooves (*P* = 0.029). The post hoc test showed the Ultra X to be significantly more effective than the PUI in CH elimination (*P* = 0.023). Comparing the root thirds in each experimental group revealed that Ultra X and EndoActivator removed significantly more CH from the coronal grooves compared with the apical ones (*P* = 0.048, *P* = 0.032, respectively). PUI activation was more effective at the coronal and middle compared with the apical third (*P* = 0.019, *P* = 0.041; respectively).

**Table 1:** The scoring results of the coronal, middle, and apical grooves

Group	Median	Interquartile range	Minimum	Maximum
*Coronal*				
Ultra X^a^	1.0	1.0	0.0	1.0
EndoActivator^a^	1.0	0.0	0.0	1.0
PUI^a^	1.0	1.0	0.0	3.0
*Middle*				
Ultra X^a^	1.0	0.0	0.0	3.0
EndoActivator^a^	1.0	0.0	1.0	2.0
PUI^a^	1.0	2.0	0.0	3.0
*Apical*				
Ultra X^a^	1.0	1.0	0.0	2.0
EndoActivator^ab^	2.0	1.0	1.0	3.0
PUI^b^	2.0	2.0	1.0	3.0

*PUI* passive ultrasonic irrigation

^ab^Ranking: statistically significant differences among Ultra X, EndoActivator, and PUI at each root canal third (*P <* 0.05)

## Discussion

The null hypothesis was partially rejected as Ultra X significantly removed more calcium hydroxide from apical grooves compared with PUI.

The residual amount of medicaments within root canals can be measured through different methods like scanning electron microscopy [30] or measurement of the surface area of the canal walls and residues [31] and volumetric analysis via micro or spiral computed tomography [32]. In area measurement, only the superficial layer of CH is considered without accurately determining the amount of CH residual on canal walls. Furthermore, computed tomography is restricted due to low availability and high cost. The present study adopted a stereo microscope with a 20× magnification and a 4-grade scoring system to evaluate and compare the efficacy of a classic PUI (28–32 kHz working frequency), EndoActivator (160–190 Hz working frequency), and Ultra X (45 kHz working frequency) in removing the CH remnants from artificial standardized grooves at coronal, middle and apical thirds, which reflected the complexity of root canals at different levels. We used a four-grade scoring system described by Lee et al. [29] and used in various previous studies [7, 10, 23, 33] for evaluation of the amount of CH removal in the grooves by two calibrated endodontic specialists. Owing to several advantages like ease of application, more reproducibility than other scoring systems, and high interexaminer agreement [4, 16], this scoring system has been widely used for CH removal evaluation in the literature [4, 7, 10, 16, 23, 33, 34]. However, this scoring system cannot accurately evaluate the CH removal in depth. In the current study, the two observers scored individually, and in case of disagreement, they discussed reaching an agreement.

EndoActivator functions based on the sonic activation of irrigants, while PUI and Ultra X are ultrasonic activation devices. Although both sonic and ultrasonic activation increases the efficiency of CH removal from root canals [35], selecting the optimal technique is a challenge [15]. Sonic devices with frequencies lower than 3 kHz generate a flow of irrigants through cavitation and acoustic streaming that clean the surfaces. The higher frequency of ultrasonic devices increases the streaming velocity of irrigants compared with sonic activation [36, 37]. Like other studies [4, 10, 16, 18, 24], the present study failed to completely eliminate CH from artificial standardized grooves in different thirds of the root canals.

The present findings showed no remarkable differences among the three groups in the coronal and middle thirds. However, in the apical third, Ultra X was significantly more efficient than the classic PUI in removing CH from the grooves. This finding was in agreement with Guven et al.'s study [27] which reported Ultra X as significantly more efficient than the other PUI device (Endosonic Blue) in removing CH from artificially created apical grooves in root canal walls. While Ultra X and EndoActivator were not significantly different in the current study, Guven [27] reported the former to be significantly more efficient in removing CH from apical grooves in root canal walls. However, it should be noted that they used EDTA before activating the devices, whereas the present study employed sodium hypochlorite. EDTA has been reported to enhance calcium hydroxide removal from the root canal walls via a chemical reaction [38–40], which justifies Guven's different findings.

Nor did the current findings show statistically significant differences between EndoActivator and PUI methods in CH removal, which is consistent with what was reported by Khaleel and Al-Ashaw [41], Faria et al. [42] and Turkaydin et al. [43]. In contrast, Li et al. [44] and Pabel and Hülsmann [4] noted that PUI removed more CH from the apical third than EndoActivator. However, controversial results can be explained by the vast variations in the volume and type of irrigants used in different studies.

In line with the literature [32, 41, 45], disregarding the removal technique, more CH residuals remained at the apical compared with middle and coronal grooves. This may be related to the apical packing of Ca(OH)_2_ during its removal [41]. Besides, lower volume of irrigants, smaller canal space, and anatomical complexities may hinder the action and circulation of irrigants in the apical third [45].

This study was limited due to assessing only standardized straight roots and missing to evaluate the efficacy of the tested methods in curved canals. Moreover, a natural root canal system can be more complicated than the artificially-created grooves in this study. Thus, the groove model might have resulted in an overestimation of the removal efficacy of the agitation devices. Another limitation of the current study was that the scoring system which was used, cannot accurately evaluate the CH removal in depth.

## Conclusions

Complete removal of CH from the artificial grooves was not achieved with any of the tested devices. Ultra X was significantly more effective than PUI only at the apical grooves.

## Data Availability

The datasets used and/or analyzed during the current study are available from the corresponding author upon reasonable request.

## Abbreviations

CH: Calcium hydroxide
PUI: Passive ultrasonic irrigation
